# The Role of MRI Findings in the Treatment of Testicular Adrenal Rest Tumors in a Child With Salt-Wasting Congenital Adrenal Hyperplasia: A Case Report

**DOI:** 10.7759/cureus.85320

**Published:** 2025-06-04

**Authors:** Tayiba Altaf, Manzoor Wani

**Affiliations:** 1 Medicine, University Hospital Southampton NHS Foundation Trust, Southampton, GBR; 2 Acute Medicine, University Hospitals Bristol and Weston NHS Foundation Trust, Bristol, GBR

**Keywords:** 21-hydroxylase deficiency, adrenal imaging, congenital adrenal hyperplasia, mri, testicular adrenal rest tumors, ultrasonography

## Abstract

Testicular adrenal rest tumors (TARTs) are benign, adrenal-like hyperplastic lesions that occur ectopically within the testes. They are most commonly observed in male patients with congenital adrenal hyperplasia (CAH), especially in those with inadequate glucocorticoid therapy and consequent poor hormonal control. Chronic elevation of adrenocorticotropic hormone (ACTH), driven by insufficient cortisol replacement, stimulates the growth of adrenal rest cells within the testes, leading to tumor formation. Although TARTs are non-malignant, their clinical significance lies in the potential for testicular dysfunction, structural abnormalities, and infertility if left undiagnosed or untreated. Early detection through imaging is essential to guide clinical management and preserve reproductive function.

We report the case of an 11-year-9-month-old boy diagnosed with the salt-wasting form of CAH secondary to 21-hydroxylase deficiency. Due to long-standing poor compliance with glucocorticoid therapy, the patient developed bilateral TARTs as a result of chronic ACTH overstimulation. Magnetic resonance imaging (MRI) of the scrotum revealed multiple well-defined, bilateral testicular masses that appeared T2-hyperintense with mild post-contrast enhancement, features consistent with TARTs.

This case emphasizes the pivotal role of MRI in the accurate diagnosis and longitudinal monitoring of TARTs in pediatric patients with CAH, particularly those with suboptimal hormonal control.

## Introduction

Testicular adrenal rest tumors (TARTs) are benign lesions arising from aberrant adrenal tissue that persists in the testes. These tumors are frequently observed in male patients with congenital adrenal hyperplasia (CAH), especially in the salt-wasting variant caused by 21-hydroxylase deficiency. Chronic elevation of adrenocorticotropic hormone (ACTH), due to insufficient cortisol replacement, stimulates the growth of these ectopic adrenal tissues, resulting in the formation of intratesticular masses [1,2]. Although TARTs are non-malignant, their clinical implications are significant, ranging from testicular atrophy and subfertility to permanent infertility and hypogonadism [3]. In recent studies, an overall TART prevalence of 40% (range: 14% to 89%) in classic patients with CAH has been found.

While TARTs are most commonly associated with CAH, they can also occur in other conditions that involve chronic ACTH elevation. These include primary adrenal insufficiency (Addison’s disease), ACTH-producing tumors (such as pituitary adenomas or ectopic ACTH syndrome), and familial glucocorticoid resistance. Rarely, TARTs may develop from ectopic adrenal tissue in the testes without any underlying endocrine disorder or following withdrawal from long-term steroid therapy due to a rebound ACTH increase.

Ultrasound (USG) is typically the first-line modality for testicular evaluation; however, it may not reliably distinguish TARTs from malignant tumors. Magnetic resonance imaging (MRI), with its superior contrast resolution and multiplanar capability, provides a more definitive characterization of testicular lesions and is crucial for follow-up imaging. In this report, we describe MRI findings in a pediatric patient with poorly controlled CAH and bilateral TARTs, highlighting the utility of MRI in early diagnosis and management.

## Case presentation

An 11-year-9-month-old boy, born from a third-degree consanguineous marriage, was diagnosed in infancy with salt-wasting CAH due to 21-hydroxylase deficiency. His disease course had been complicated by poor medication compliance, leading to six to eight documented adrenal crises. At 6.5 years of age, he exhibited signs of gonadotropin-dependent precocious puberty, including advanced bone age and rapid growth, ultimately compromising his predicted adult height.

In mid-2023, the patient presented with new clinical concerns, including worsening facial hair growth, emotional instability, decreased linear growth velocity, and irritability. His parents also noted bilateral scrotal swelling that had gradually increased over the preceding six months. Physical examination confirmed bilateral testicular enlargement with firm intratesticular masses.

Ultrasound

**Figure 1:**
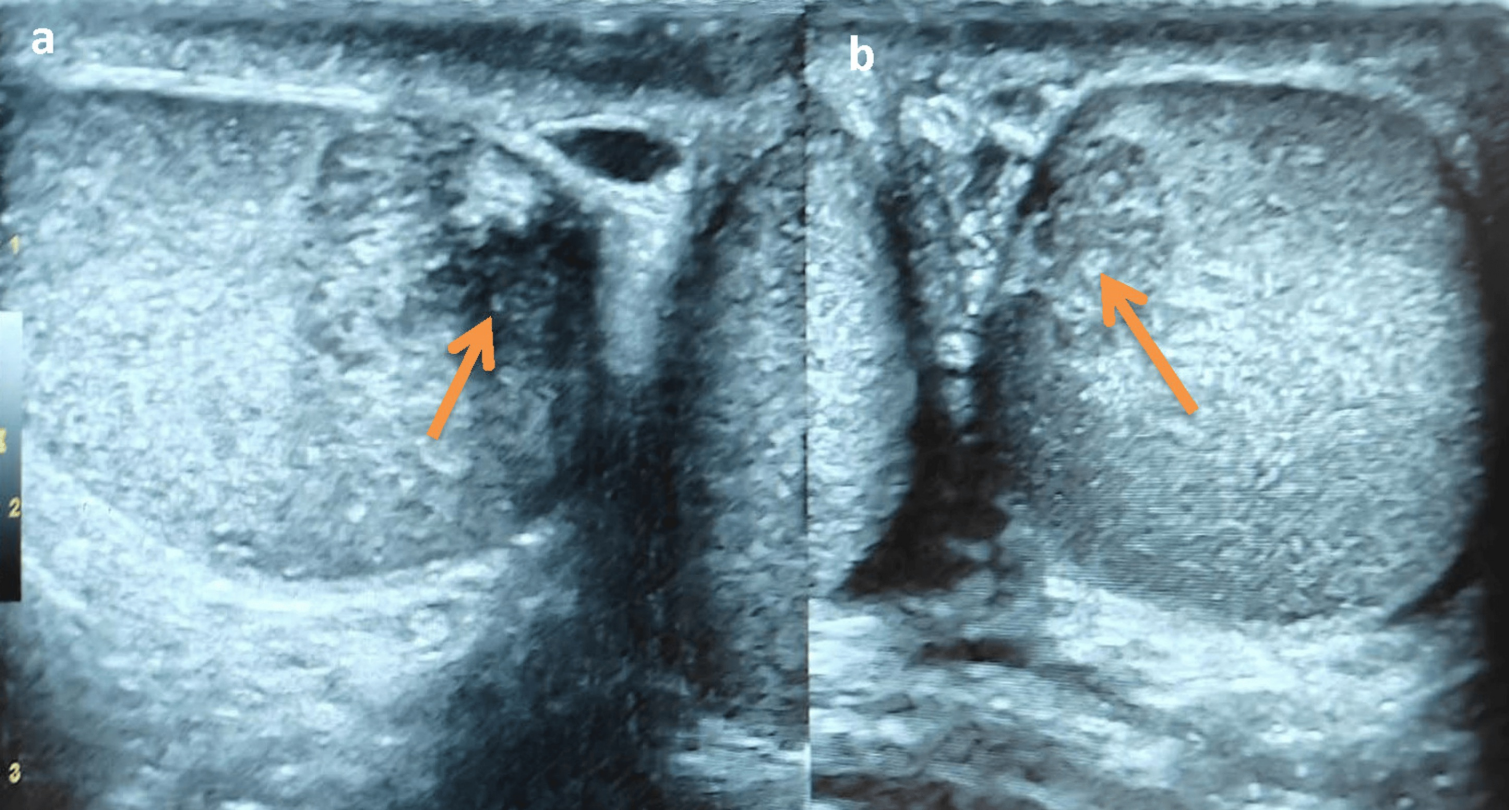
(a and b) USG showing evidence of well-defined eccentric heterogeneously hypoechoic masses with hyperechoic areas within bilateral testis (predominantly around the mediastinum of the testis). USG: Ultrasound

USG findings showed bilateral testes demonstrating the presence of well-defined, eccentric, heterogeneously hypoechoic masses, predominantly located around the mediastinum of the testis (Figure 1). Within these masses, focal areas of increased echogenicity were also noted. Colour Doppler imaging revealed no significant internal vascularity within these lesions.

Magnetic resonance imaging

**Figure 2:**
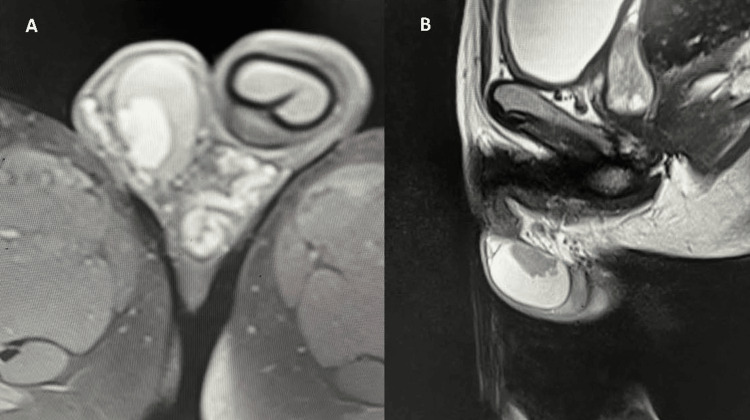
(A) T1 post-gadolinium MRI image showing post contrast enhancement; (B) T2 showing hypointense lesions and T1 hyperintensity indicating the presence of lipid-rich or proteinaceous material, which is common in adrenal rest tissue due to its steroidogenic nature. T2 hypointensity is usually due to fibrosis, compact cellular architecture, and low water content, features often seen in chronic or hormonally suppressed TARTs. TARTs: Testicular adrenal rest tumors

MRI of the scrotum revealed multiple altered signal intensity areas within the bilateral testes, primarily located around the mediastinum testis (Figure 2). T1-weighted images demonstrated iso- to hyperintense signal characteristics relative to the normal testicular parenchyma. T2-weighted images showed these lesions to be predominantly hypointense. Diffusion-weighted imaging (DWI) did not reveal any areas of diffusion restriction. Post-contrast T1-weighted images revealed avid and homogeneous enhancement of the lesions following the administration of a gadolinium-based contrast agent.

Interpretation

The imaging findings are characteristic of TARTs, particularly given the bilateral location, eccentric distribution around the mediastinum testis, heterogeneous echotexture on ultrasound, and the specific signal characteristics and enhancement patterns on MRI. The absence of diffusion restriction further supports the benign nature of these lesions. Lack of significant internal vascularity on Doppler imaging is also in keeping with typical TART findings.

## Discussion

TARTs are frequently encountered in male patients with poorly controlled CAH, with reported prevalence rates as high as 94% in adults and around 21-33% in pediatric populations [4]. These lesions originate from adrenal rest cells that migrate with the gonads during embryogenesis. Under conditions of chronic ACTH stimulation, as in untreated or poorly managed CAH, these rests undergo hypertrophy and hyperplasia, resulting in mass formation [5].

Clinically, TARTs may remain asymptomatic or present with testicular pain or enlargement. However, even asymptomatic tumors can compress seminiferous tubules, leading to impaired spermatogenesis and testicular atrophy [6]. TARTs are most commonly seen in male patients with CAH, with prevalence in this group ranging widely from 0% to 94%. While the exact number of reported cases in the literature isn't clear, TARTs are well-documented through numerous studies and case reports. Ultrasound is the most commonly used imaging tool for diagnosis due to its accessibility, while MRI is used less frequently-typically reserved for unclear cases or detailed pre-surgical evaluation

Imaging plays a pivotal role in TART identification and differentiation. While ultrasonography is widely accessible and effective for initial screening, its ability to characterize lesion composition and vascularity is limited. MRI, in contrast, offers detailed visualization of soft tissue planes, allowing for confident discrimination between benign and malignant testicular masses. In this case, the absence of necrosis, restricted diffusion, or aggressive invasion on MRI supported the diagnosis of TARTs.

Histological confirmation is rarely necessary unless the lesion is atypical in appearance or unresponsive to optimized hormonal therapy. The mainstay of treatment involves intensifying glucocorticoid therapy to suppress ACTH stimulation and promote tumor regression. Our patient was initiated on nighttime dexamethasone to target nocturnal ACTH peaks, along with physiological hydrocortisone and fludrocortisone replacement.

Follow-up MRI is planned in six months to assess tumor regression. Regular endocrinological and urological monitoring is essential to assess hormonal control and testicular function. Fertility preservation strategies should also be discussed as the patient nears adolescence.

## Conclusions

This case highlights the importance of high-resolution MRI in the evaluation of testicular masses in pediatric CAH patients. MRI not only confirms the diagnosis of TARTs but also differentiates them from malignant entities, guiding appropriate therapeutic decisions. Early radiological identification and effective hormonal suppression can prevent long-term sequelae, including infertility and testicular atrophy. Given their high prevalence and potentially reversible nature, TARTs should be routinely screened for in male patients with CAH, especially those with suboptimal hormonal control.
